# Responding effectively to adult mental health patient feedback in an online environment: A coproduced framework

**DOI:** 10.1111/hex.12682

**Published:** 2018-04-06

**Authors:** Rebecca Baines, John Donovan, Sam Regan de Bere, Julian Archer, Ray Jones

**Affiliations:** ^1^ Collaboration for the Advancement of Medical Education Research and Assessment University of Plymouth Plymouth UK; ^2^ Volunteer Mental Health Patient‐Research‐Partner Plymouth UK; ^3^ School of Nursing and Midwifery University of Plymouth Plymouth UK

**Keywords:** coproduction, justice theory, mental health, patient feedback, quality improvement, response framework

## Abstract

**Background:**

Responding to online patient feedback is considered integral to patient safety and quality improvement. However, guidance on how to respond effectively is limited, with limited attention paid to patient perceptions and reactions.

**Objectives:**

To identify factors considered potentially helpful in enhancing response quality; coproduce a best‐practice response framework; and quality‐appraise existing responses.

**Design:**

A four‐stage mixed methodology: (i) systematic search of stories published on Care Opinion about adult mental health services in the South West of England; (ii) collaborative thematic analysis of responses to identify factors potentially helpful in enhancing response quality; (iii) validation of identified factors by a patient‐carer group (n = 12) leading to the coproduction of a best‐practice response framework; and (iv) quality appraisal of existing responses.

**Results:**

A total of 245 stories were identified, with 183 (74.7%) receiving a response. Twenty‐four (9.8%) had been heard but not yet responded to. 1.6% (n = 4/245) may lead to a change. Nineteen factors were considered influential in response quality. These centred around seven subject areas: (i) introductions; (ii) explanations; (iii) speed of response; (iv) thanks and apologies; (v) response content; (vi) signposting; and (vii) response sign‐off that were developed into a conceptual framework (the Plymouth, Listen, Learn and Respond framework). Quality appraisal of existing responses highlighted areas for further improvement demonstrating the framework's utility.

**Conclusion:**

This study advances existing understanding by providing previously unavailable guidance. It has clear practical and theoretical implications for those looking to improve health‐care services, patient safety and quality of care. Further validation of the conceptual framework is encouraged.

## 1 INTRODUCTION

Patient feedback is considered integral to quality improvement and patient safety.^1^, ^2^, ^3^, ^4^ The advent of Web 2.0 and subsequent electronic word‐of‐mouth (eWOM) platforms such as Patient Opinion (now Care Opinion) (http://www.careopinion.org.uk) and iWantGreatCare (http://www.iwantgreatcare.org) has transformed not only the ways in which patients access and evaluate health‐care services, but also the way in which they publically share their health‐care experiences.^5^, ^6^, ^7^ However, in spite of their acknowledged importance and increasing use,^8^ limited attention has explored how health‐care organizations respond to patient feedback online, how patients perceive and react to these responses, and how organizational responses might be improved.^5^

Being able to effectively respond to patient feedback is considered important if health‐care providers are to better monitor patient safety and quality of care,^1^ improve systemic issues and encourage patient‐centred care.^4^, ^5^, ^9^, ^10^ As described by Doig and others, it is possible to complete a feedback process with a higher opinion of the organization if the feedback process has been satisfactory.^5^, ^9^ In contrast, the provision of an unsatisfactory response can lead to negative emotions including frustration and dissatisfaction.^9^, ^11^ While some patients may accept that service provision can go wrong due to human error, as suggested by Rio‐Lanza, an organization's response, or indeed lack of response, to the service failure can be the most likely cause of service dissatisfaction.^10^ Understanding factors that can help facilitate effective organizational responses is therefore imperative.^10^, ^12^

In opposition to medical or health‐care service literatures which have typically taken a procedural and epidemiological view of feedback processes, other literatures such as those from business and hospitality disciplines have developed a significant body of research.^5^ Such literatures indicate that organizational responses can have profound implications for public inferences of trust, perceived responsiveness, organizational reputation, customer satisfaction and further complaint behaviour.^9^ One theory often applied in business and hospitality literatures to understand response dissatisfaction is perceived justice, or justice theory.^10^, ^12^, ^13^ Based on the premise that perceptions of organizational responses influence satisfaction and future behavioural intentions,^12^ justice theory is a multifaceted construct encompassing three dimensions: procedural, interactional and distributive justice. Procedural justice refers to the perceived fairness of policies and procedures used by the responding organization with response waiting times, accessibility and perceived efficiency considered particularly important. Interactional justice focuses on the manner in which individuals are treated during the response process, for example with courtesy, respect, honesty and assurance, while distributive justice relates to the perceived fairness of the outcome offered by the responding organization such as compensation.^10^, ^13^ As described by Blodgett et al and others, justice theory is considered a valuable framework for understanding reactions to organizational responses.^10^, ^12^, ^13^ However, it is yet to be applied in a health‐care environment that specifically explores patient reactions to online responses.

Informed by principles of collaborative working,^14^ this research sought to explore patient reactions to existing organizational responses leading to the development of a coproduced conceptual framework. It advances existing understanding by moving beyond complaints as historically researched,^5^ avoiding a “top‐down” approach by collaborating with a volunteer mental health patient‐research‐partner and wider patient‐carer support group (Heads Count; http://www.colebrooksw.org/heads-count/), and exploring patient response reactions from a population frequently described as “seldom heard”—mental health.^15^, ^16^, ^17^

For brevity, the term “patient” is used to be inclusive of service users, customers, clients, consumers, carers and/or family members, although the important distinctions between these terms are acknowledged. For clarity, we have used “response” to mean an organizational response and “stories” to mean feedback provided by patients.

## 2 METHODS

### 2.1 Design

We used a mixed‐methodology approach comprised of four interrelated stages. Firstly, adult mental health stories published on one of the United Kingdom's leading patient feedback websites, Care Opinion (previously Patient Opinion), were identified through a systematic search. Secondly, a representative sample (20%, n = 37) of identified responses from the initial sample were thematically analysed using an inductive approach in collaboration with a volunteer patient‐research‐partner to identify factors potentially helpful in enhancing response quality. Thirdly, factors considered influential were discussed and refined by a wider patient‐carer stakeholder group (n = 12), Heads Count, leading to the coproduction of a best‐practice response framework. Finally, existing responses were quality‐appraised using the developed framework.

Due to resource constraints and the large number of patient stories published on Care Opinion at time of publication (over 190 345), only stories and corresponding responses made in the South West of England were included.

Care Opinion was selected as the database for this research due to its ability to directly respond to patient feedback through a dialogue exchange and its high number of responding organizations, over 600 at the time of publication. The focus on a single website such as TripAdvisor, of which Care Opinion shares some similar functions, has been adopted in other research studies.^18^

Care Opinion works on the premise that (i) patients share their story, (ii) the story is sent to relevant staff members to facilitate learning, (iii) patients receive a response, and (iv) the original patient story may lead to a beneficial change. On publication, staff members in subscribing organizations who have opted into alerts are made aware of the story. Other relevant organizations are also contacted by Care Opinion. A responder may indicate in their response that they have made a change as a result of the feedback received. This claim is made by the responder and not Care Opinion. A self‐reported change is then visually shown on the website. It is up to individual or organizational discretion who responds. There is no guarantee that patients will get a response. All stories and subsequent responses published on Care Opinion are publically available providing real‐time feedback with the intention of providing cost‐effective, measurable and transparent improvements.

Responses to adult mental health stories were selected because of the acknowledged difficulties in satisfactorily responding to this population. Mental health is often reported as one of the most problematic areas to obtain, and respond to, patient feedback due to acknowledged trust issues and low response rates.^19^ O'Regan and Ryan suggest that exploring patient feedback in mental health is of paramount importance as patients are more likely to maintain contact with medical services if they are satisfied with their care.^20^ This, in turn, has implications for reducing clinical relapse incident and hospital admission rates affecting patient well‐being and resource expenditure.^20^

#### 2.1.1 Search strategy

Stories about adult mental health services or experiences in the South West of England published by Care Opinion from its inception in January 2005 to 25 January 2017 were systematically searched using the following search terms: “mental health” OR “mental illness” OR “mentally ill” OR “mental” OR “pnd” OR “psychiatrist” OR “psychiatry” OR “depression” OR “anorexia” OR “anxiety” OR “eating disorder” OR “psychology” OR “psychosis” OR “psychotic” OR “ptsd” OR “self‐harm.” Search terms were designed using the Peer Stories of Electron Search Strategies (PRESS) guidance^21^ in collaboration with the patient‐research‐partner and CEO of Care Opinion to maximize sensitivity and specificity.

#### 2.1.2 Data selection

One reviewer independently screened all identified stories using a piloted inclusion criteria form to ensure inclusion/exclusion standardization. To maintain accuracy, a representative sample (20%, n = 37) was also screened for inclusion by the patient‐research‐partner. Any discrepancies were resolved by discussion with a third research team member where needed.

#### 2.1.3 Inclusion and exclusion criteria

Only stories that discussed the treatment or diagnosis of a mental health condition, experience or service were included. Stories that did not achieve this were excluded. Exclusion examples include being anxious about a tooth removal operation.

#### 2.1.4 Data analysis

Data analysis was conducted in three interrelated stages:

1. An inductive thematic analysis of response content by the patient‐research‐partner and first author to collaboratively identify factors considered potentially helpful in a response.^22^ Due to the originality of this research, a deductive approach that imposed pre‐defined categories may have restricted novel knowledge generation and was not therefore suitable for the purposes of this research.
2. Identified factors were refined and validated by Heads Count, a local mental health patient‐carer support group (n = 12), through a round‐table discussion. This was audio‐recorded and transcribed verbatim by the first author. During the two‐hour discussion chaired by a Heads Count member, the first author and patient‐research‐partner facilitated group discussion following the presentation of the representative sample (n = 37) reviewed by the patient‐research‐partner and identified factors in stage 1. After minor refinements to the wording of factors, the patient‐carer group and patient‐research‐partner organized factors into groups with minimal professional input. This process was facilitated by individually listing agreed factors onto Post‐it notes and organizing them into logical groups accordingly. The framework is presented in the order agreed by participants. No new factors were suggested by participants at any stage.
3. The validated framework was then used to quality‐appraise existing responses by the first author and patient‐research‐partner to determine how existing responses aligned themselves to patient perceptions and reactions.

## 3 RESULTS

### 3.1 Stage 1: story identification

From the 190 345 stories published on Care Opinion at the time of analysis, 2386 (1.25%, 2386/190 345) were identified as stories about adult mental health services in England. A total of 245 (10.3%, 245/2386) were from the South West and analysed for the purposes of this research.

**Figure 1:**
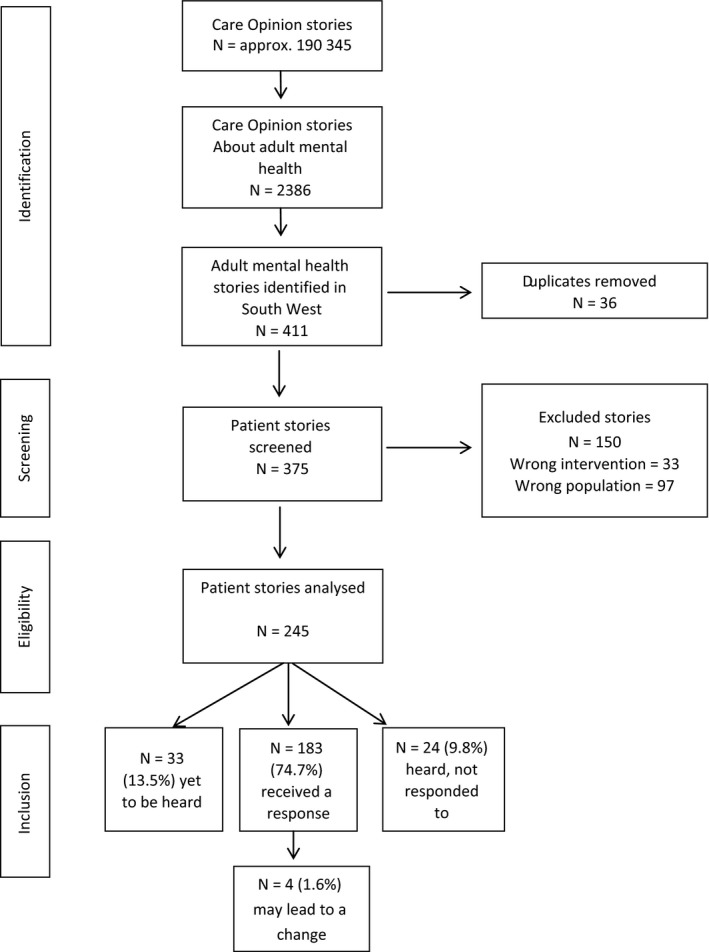
Inclusion process of patient stories published on Care Opinion about adult mental healthcare services or experiences in the South West of England

A total of 183 (74.7% 183/245) received a response from 41 different job roles or titles (see supplementary material). Thirty‐eight (15.5%) stories were yet to be heard by a subscriber, and 24 (9.8%) had been heard but not yet responded to at the time of analysis. Only 1.6% (n = 4/245) of included responses were tagged by the organization as “may lead to a change.” The inclusion process is shown in Figure 1.

11% (n = 27/245) of identified stories received multiple responses. For clarity, only the first response was included for analysis.

### 3.2 Stage 2: thematic analysis

Collaborative thematic analysis identified 19 factors as potentially helpful in enhancing organizational response quality. Some factors were considered only applicable to positive and/or negative stores. These are indicated in Table 1.

**Table 1:** Factors identified as potentially influential in enhancing response quality

1. Provides a photograph of responder	11. Offers reassurance^b^
2. Provides responder name	12. Tailors response
3. Names the story provider in response	13. Offers to make contact with the story provider at a later date^b^
4. Identifies responder role	14. Signposts patient to other relevant services^b^
5. Provides explanation of responder role	15. Explains purposes of signposted services and why these have been suggested^b^
6. Explains why the responder in particular is responding	16. Provides contact details *and* a named person for these services^b^
7. Responds within 7 days	17. Provides opening times for suggested services^b^
8. Offers thanks for providing patient story	18. Suggests more than 1 contact option^b^
9. Offers to pass feedback onwards if positive in nature^a^	19. Signs off response in a polite manner
10. Provides an apology^b^	

a Only applicable to positive/mixed stories.

b Only applicable to negative/mixed stories.

### 3.3 Stage 3: validation of influential factors by stakeholder group

Factors considered influential by the patient‐research‐partner were reviewed and refined by a patient‐carer stakeholder group (n = 12). During this stage, no new factors were suggested by participants. Only minor revisions to factor wording were suggested. Agreed factors primarily centred around seven subject areas: (i) introductions; (ii) explanations; (iii) speed of response; (iv) thanks and apologies; (v) response content; (vi) signposting; and (vii) response sign‐off. Each subject area and its corresponding factors are discussed in turn below.

#### 3.3.1 Introductions

Introduction through the provision of a responder's picture, name and role was considered essential. This was seen as a useful triad of information. Failure to do so was perceived as particularly problematic “*as it is hard to forge a trustful relationship with someone without knowing their name*” (Heads Count patient‐carer member [from here on H.C. member] 3). Participants wanted to know “*who they are talking to*” and “*what their position is*” (H.C. member 1), considering this as “*standard good manners*” (patient‐research‐partner). Introductions were considered “*really important*” (H.C. member 2), particularly if the patient had experienced a negative encounter.

#### 3.3.2 Explanations

Explanation of the responder's role was also considered important due to perceived complexity of health‐care services and importance of introductions mentioned above.

#### 3.3.3 Speed of response

The provision of a timely response was considered pivotal. A response within 7 days was deemed acceptable by the mental health patient‐research‐partner and H.C. members, although a response within 3 days was considered desirable. Anything beyond these timescales was considered to hold important implications for the reputation, perceived responsiveness and sensitivity of organizations concerned.

#### 3.3.4 Thanks and apologies

Thanking patients for taking the time to write their stories was considered imperative irrespective of story content, that is positive or negative. Some terminology was deemed more favourable than others. For example, the phrase “thanks” was considered “*almost sarcastic*” (patient‐research‐partner), while “thank you” appeared more sincere. In spite of this, the presence of a “thank you” was considered influential in patient response satisfaction by all participants.

Sharing positive feedback with those involved was also considered a central feature of patient response satisfaction. This was identified as one of “*the sole reasons behind patients taking the time to provide feedback—it must get back to them, it must make a change, otherwise what's the point?*” (H.C. member 3).

In partnership with thanking story providers, offering an apology was also considered imperative, particularly if the patient had experienced a negative or mixed encounter or a significant delay in response times.

#### 3.3.5 Response content

Tailoring response content was considered important: “*the last thing you appreciate is a typed written response; you want to be treated as an individual, given an individual response*” (H.C. member 4). Participants suggested patients are quick to detect “*standardized*” or “*meaningless*” responses (patient‐research‐partner). When examining organizational responses as part of the round‐table discussion, one H.C. member stated: “*it's a couldn't care less response, in my tray and out again, typical standard, makes you question what's the point? It's not going anywhere?*” (H.C. member 7). In one of the examples reviewed, the original patient reviewer responded to the organization stating: “*thank you for your automated response*” (Care Opinion identifier 164520 from here on COI). H.C. members suggested patients require assurance or “*evidence that they've* [the responder] *read the stories content*” (H.C. member 6).

#### 3.3.6 Signposting

A further core function of responses identified was the signposting of other services. However, the assumption of patient awareness and understanding of such services was identified as particularly problematic by participants. For example*,* “*not many people know about PALS* [Patient Advice and Liaison service]*, will they take it up?*” (H.C. member 9).

H.C. members also identified a critical need for responders to provide a specified contact name, opening times and multiple contact options for signposted services as phone calls can induce anxiety, particularly “*when you don't even know the name, or role, of the person you're supposed to be ringing*” (H.C. member 12). Essentially forum members wanted to know “*how do I contact you—phone, email other means*?” (H.C. member 10): something considered necessary to enable an “*accessible*” (H.C. member 2) dialogue between patients and a service.

#### 3.3.7 Sign‐off

Finally, the phrasing of the sign‐off used at the end of a response, for example, best wishes' and kind regards, was considered important. The ultimate question the patient‐research‐partner and stakeholder participants wanted responders to ask themselves before response submission was “would you be happy receiving this response?”

#### 3.3.8 Framework development

**Figure 2:**
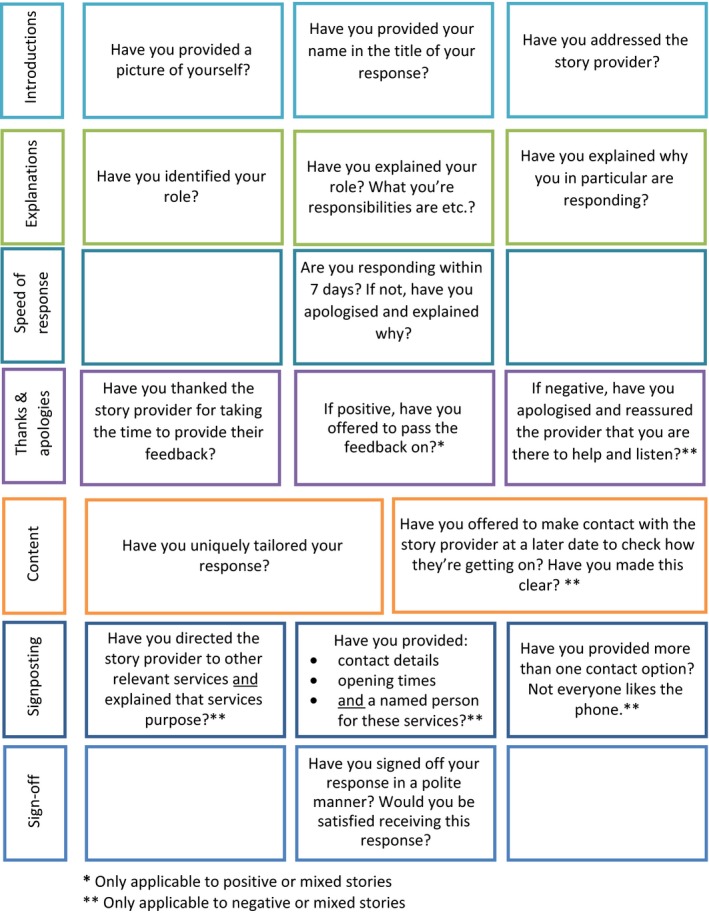
The coproduced Plymouth Listen, Learn and Respond framework

The organization of agreed factors during the round‐table discussion led to the codevelopment of a best‐practice response framework entitled the “Plymouth Listen, Learn and Respond framework” (PLLR) presented in Figure 2. Through this process, factors 10‐11, 14‐15 and 16‐17 were combined to encourage ease of use and understanding.

### 3.4 Stage 4: quality appraisal of existing responses

**Figure 3:**
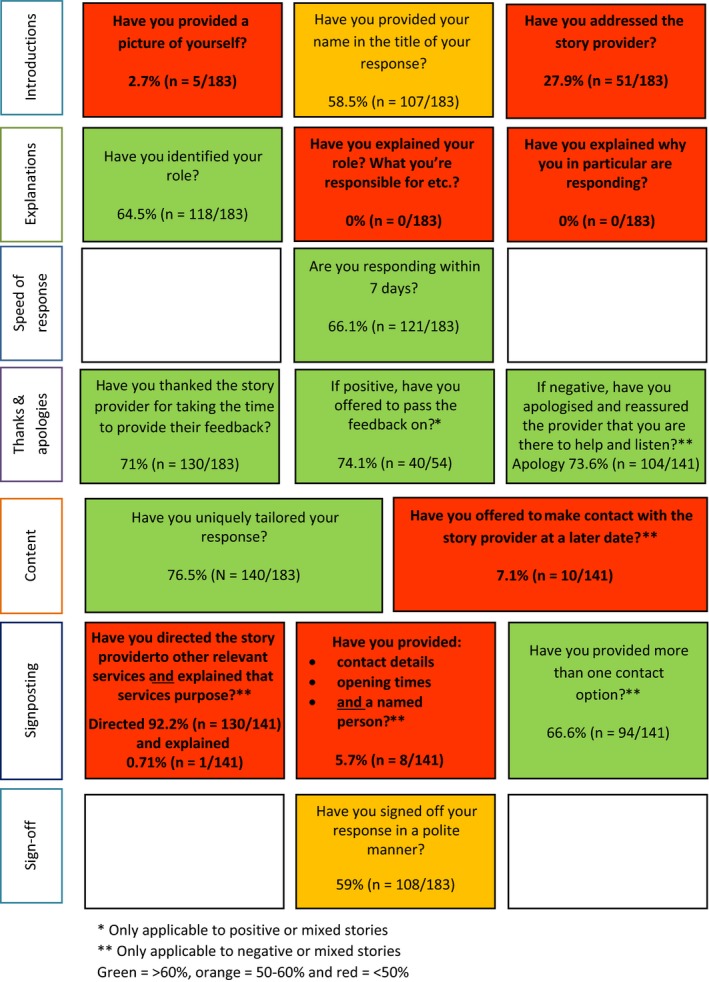
Quality appraisal of existing responses showing areas of good practice and room for improvement

Quality appraisal of existing responses using the agreed framework indicated a need for improvement in providing a picture of the responder; addressing the story provider; explaining the responders role; explaining why they in particular are responding; offering to make contact with the provider at a later date; directing the provider to relevant services *and* explaining the purposes of these services; and providing contact details, opening times and a named contact for signposted services. A “traffic light” colour coding system (green ≥ 60%; orange = 50%‐60%; and red ≤ 50%) shown in Figure 3 is used to denote areas of good practice and room for improvement. Results are discussed in the same seven subject areas as the preceding stage.

#### 3.4.1 Introductions

27.9% (n = 51/183) of responses directly addressed the story provider (factor 3; Table 1), for example “Dear Anonymous” (COI 177411). Less than 3% (n = 5/183, 2.7%) of responders provided a picture of themselves, their name and role (factors 1‐3; Table 1). This may be due to Care Opinion historically not having picture provision as an option. However, where provided, this was positively experienced by participants, for example “*that's nice, she's put a face to the name, nice smile, looks friendly, not like she's going to jump down your throat in uniform*” (H.C. member 3).

Over half of responders provided their name (n = 107/183, 58.5%), with over two‐thirds of responders also providing their role (n = 118/184, 64.5%) (factors 2 and 4; Table 1). However, 41 different responding identities were reported (see supplementary material). A responder themselves admitted the complexity of various roles and health‐care systems: “*I appreciate how confusing these systems can appear*” (COI 224190).

In some cases (n = 3), the responder title did not match the response's content. For example, in one instance, the response was titled as Head of Engagement and Responsiveness, but then completed by the Clinical Director of adult mental health (COI 27919).

#### 3.4.2 Explanations

Despite the variability of roles identified (n = 41), no responder provided an explanation of their role, or explained why they in particular were responding (factors 5 and 6; Table 1).

#### 3.4.3 Speed of response

Response times significantly varied with responses ranging from 0 to 1412 days (M = 31.2, SD = 145.1). When two outliers of 1412 and 1208 days were included, the mean average response rate was 31.2 days. When removed, the mean average decreased to 17.1 days (range = 585, M = 17.1, SD = 52.8). Although many organizations offered reassurance (factor 11; Table 1) that “*we take your comments very seriously*” (COI 168497), and for patients to be “*assured that we are listening to you*” (COI 198899), such assurances became “undone” if organizations failed to respond in sufficient time. However, nearly half (49.7%, n = 91/183) of responders responded within 3 days of story publication. 66.1% (n = 121/183) responded within 7 days. Response times were considered important for the reputation, perceived responsiveness and sensitivity of services concerned. For example, “*I have concluded the Trust does not seem to want to use service users feedback to provide a better service, I feel they have denied my complaint instead of using valuable feedback to gain improvements in bettering services for people in this area*” (COI 255461).

#### 3.4.4 Thanks and apologies

71% (n = 130/183) of responders thanked patient reviewers: “*Thank you for taking the time to…*” (COI 27507), and “*thank you for having the courage to do so*” (COI 230306). 73.6% (n = 104/141) of responders also offered an apology (factor 10). Some (40.4%, n = 42/104) adopted inclusive pronoun use, that is “*we are very sorry to hear…*” (COI 80348), while the majority (58.65%, n = 61/104) of responders accepted more individualized responsibility, that is “*I'm very sorry to hear…*” (COI 222703). One responder adopted the pronoun “our.” Additional reasons for apologies included not being able to offer a more detailed response, and for delays in response times “*I apologise for the delay in responding but I had to have my access rights reset after some leave*” (COI 308835).

Where applicable (positive, n = 43; mixed, n = 11), 74.1% (n = 40/54) of responders offered to forward on positive feedback, a central feature of patient response satisfaction (factor 9; Table 1). For example, “*Dear Doricigirl, thank you for your kind comments…. As requested, I have fed back your comments to the Maternity Teams…*” (COI 321922). Staff morale and learning benefits associated with this action were also highlighted by several responders: “*Thank you so much for taking the time to give your positive feedback…. I will pass your thoughts on to the staff… as it means a lot to staff when people appreciate their efforts and hard work*” (COI 157625); and “*We always welcome comments regarding experiences… as it helps us to identify problems and improve the quality of our service and care*” (COI 139286). However, one identified limitation of the valued anonymization of patient stories was the inability to pass on positive praise in line with patient wishes. For example, “*We can share your thanks with staff members directly involved, if you would like us to do this, we will need a little more specific information from you*” (COI 173372).

#### 3.4.5 Response content

**Figure 4:**
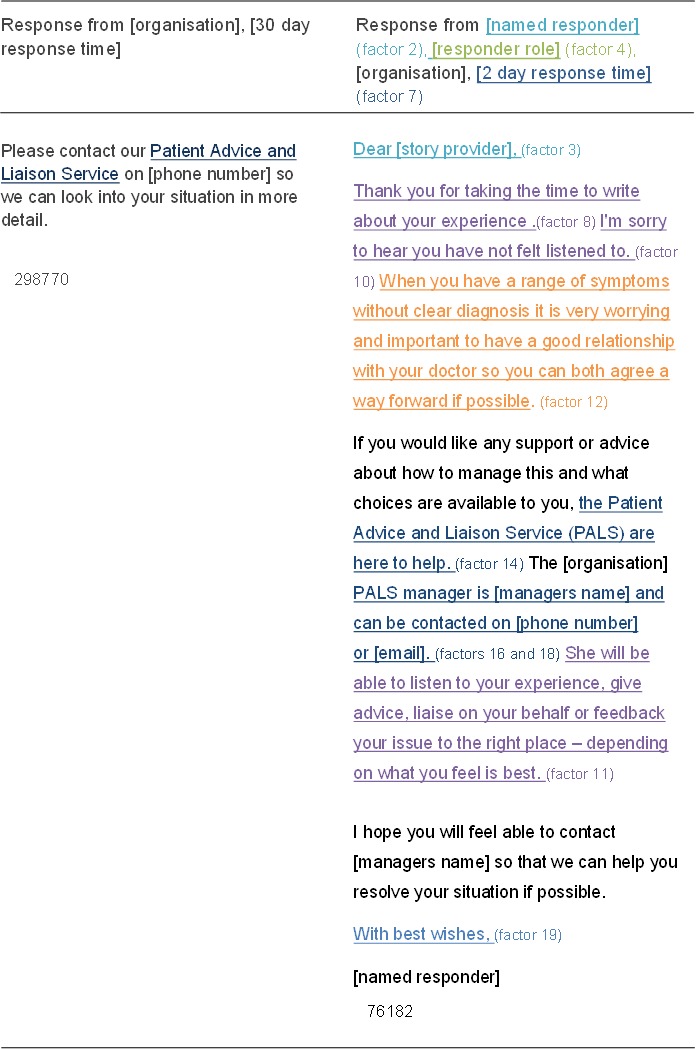
Comparison of standardized vs tailored response as assessed by patient participants

76.5% (n = 140/183) of responses appeared to tailor their response. Figure 4 provides an example of a “*standardized*” and tailored response perceived as particularly poor/good by participants. These fulfil 1/18 and 12/18 factors, respectively, due to their negative categorization, that is factor of offers to pass positive feedback on not applicable. Fulfilled factors are colour‐coordinated with the seven subject areas identified in Figure 2.

#### 3.4.6 Signposting

Over two‐thirds (92.2%, n = 130/141) of responses signposted other services (factor 14; Table 1). This was primarily the Patient Advice and Liaison Service (PALS) (73.9%, n = 96/130). However, only one responder provided an explanation for the proposed service (factor 15; Table 1), with many assuming existing patient awareness and understanding. However, some areas of good practice are clearly demonstrated (COI 206461). Furthermore, the efficiency and suitability of PALS was questioned by many patient reviewers. Ensuring signposted service efficiency is therefore also key to maintaining patient response satisfaction.

Closely aligned to signposting relevant services was the provision of opening times, multiple contact options and named personnel. Of the relevant responses analysed (negative, n = 129; mixed, n = 11), 66.6% (n = 94/141) provided more than one contact option, primarily telephone numbers and email addresses (factor 18; Table 1). However, less than one‐fifth of responses provided a named person and their corresponding contact details (17.8%, n = 25/141), a criterion deemed essential from a patient perspective (factor 16; Table 1). Even fewer (5.7%, n = 8/141) provided opening times for signposted services (factor 17; Table 1).

#### 3.4.7 Sign‐off

Finally, over half of responses were considered to be signed off in a polite manner (59%, n = 108/183), for example “*Best wishes*” (COI 248529); “*with kind regards*” (331704); and “*thank you once again*” (COI 173372).

## 4 DISCUSSION

This research advances existing understanding by providing previously unavailable guidance on how to effectively respond to patient feedback in an online environment.^5^, ^6^, ^23^, ^24^ Informed by principles of collaborative working,^14^ this research identified 19 factors considered influential in effective organizational responses from a patient perspective. It identifies effective responses as those that address the feedback provider; introduce the responder through the provision of their name, role and picture; provide an accessible explanation of their role; offer thanks and apologies where appropriate; respond within seven days; and provide uniquely tailored response content that includes a named contact, opening times and multiple contact methods for signposted services.

Quality appraisal of existing responses identifies a clear need for existing response methods to be refined. For example, providing contact details, opening times and a named person contact for signposted services occurred in less than 6% of responses reviewed despite their perceived importance from a patient perspective. Research findings indicate a current misalignment between patient aspirations and response practice, helping to explain previous reports of patient response dissatisfaction.^5^

When discussed in line with justice theory, a multifaceted construct encompassing three dimensions frequently used in business and hospitality sectors to understand feedback processes,^5^, ^6^ a theoretically robust understanding of reported results can be developed. For example, procedural justice refers to the perceived fairness of policies and procedures used by responding organizations of which the concept of voice and neutrality appears key. Similar to consumers, patients appreciate the opportunity to have their voice heard.^5^ The rapid growth of eWOM platforms such as Care Opinion and others globally provides such an opportunity. However, their associated benefits can be restricted by problems associated with neutrality—the degree to which their processes appear scripted or “standardized.”^5^, ^6^ As demonstrated by participants in this research, patients are quick to detect “*standardized*” or “*meaningless*” responses, often leading to feelings of frustration and dissatisfaction. The patient‐generated criterion reported in this study of providing a uniquely tailored response that demonstrates that the responder has actively listened to a patient's experience is a direct response to this unfavourable approach. Other aspects of procedural justice relate to timely response efforts. Our research highlights the importance of rapid responses (within seven days, although three is desirable) in facilitating favourable perceptions of organizations, perceived responsiveness and sensitivity of organizations concerned.

The second dimension of justice theory relates to the manner in which individuals are treated during the response process (interactional justice).^5^ Our research strongly suggests responses need to provide appropriate explanations, be made accessible to patients and be presented in a polite, empathic manner facilitated by assurance, honesty and respect. Due to the acknowledged importance of communication in organizational responses,^13^ the concept of interactional justice appears particularly relevant to understanding response perceptions and reactions.^12^ Finally, distributive justice refers to compensation evaluation.^5^ In most instances, feedback related to health‐care services is considered “unrecoverable”; that is, simply reperforming the service is not possible.^5^ Reasons behind patient feedback submission in this context often therefore involve more egocentric exchanges such as apologies or reassurance.^5^ Contrary to medicolegal concerns,^5^ none of the patient stories reviewed referred to monetary compensation.^5^ Stories reviewed typically focused on more egocentric compensations such as service failure prevention for other patients, and improving existing services for others. These may be important motivators to consider when trying to encourage patients to share their feedback. Other egocentric motivations identified also importantly included the forwarding‐on of positive patient feedback expressing praise and gratitude for services received—a desirable and motivational aim often overlooked in existing research. The beneficial impacts of receiving positive feedback should not be undermined.

Following this, our research findings raise important practical and theoretical implications. Firstly, why less than 2% of patient stories may lead to a possible change warrants further investigation, as does the high proportion of stories not yet responded to at the time of analysis. Patient feedback can only be acted on if it is acknowledged and heard. As stated by Coulter et al, to ignore patient experience data, particularly when asked, is unethical.^25^ Continuing to ignore such information would only be to the detriment of patient safety and quality of care. As acknowledged in the difficulties of forwarding on positive feedback, it remains unclear whether a lack of self‐reported change is related to problems in data collection methods, for example detail specificity, or wider professional and organizational cultural issues that inhibit patient feedback acceptance and subsequent action.^26^, ^27^ If it is an issue of nuanced detail, then collection methods and their structures/guidance may need to be revised to facilitate this process. Alternatively, if it is an issue of culture, then ways of resolving this are needed as the sharing of patient experience is becoming an inevitable component of quality improvement internationally.^1^, ^2^, ^3^, ^4^ Other implications of this research include the possibility to enhance response quality and subsequent quality improvement initiatives by following the collaboratively designed “Plymouth Listen, Learn and Respond” (PLLR) framework. This in turn may help to diffuse patient anger, prevent further complaint behaviour and alleviate any immediate sense of injustice caused.^9^ Further research into this area is desirable. While designed for online environments, identified factors could also apply “offline,” although further investigation into this issue is required. Importantly, identified areas for improvement are considered “*fixable and not insurmountable*” (H.C. member 8). Organizations and individual practitioners should therefore be able to strengthen their response quality with relative ease. While factors identified as influential may represent limited associated costs, the power of high‐quality responses to act as drivers for organizational learning, quality improvement and patient empowerment should not be underestimated. As one Care Opinion reviewer states: “*I felt empowered and understood and believed and respected…which is all I ever needed in the first place*” (182221). Commitment to strengthening response processes is therefore actively encouraged.

### 4.1 Strengths and limitations

Strengths of this research include its application of a systematic search; inclusion of online feedback; development of an innovative response framework in collaboration with a patient‐research‐partner and patient‐carer group; inclusion of a seldom‐heard population—mental health; and unique application of justice theory to understand online responses in a health‐care setting. Extensive patient involvement throughout the process revealed unique insight into patient reactions and perceptions of organizational responses. Without such involvement, factors identified as influential may have continued to be under‐reported and therefore excluded in future quality improvement developments. For example, the importance of providing more than one contact option due to the anxiety involved in phoning an unnamed individual raised by the patient‐research‐partner and others may not be something at the forefront of professional minds with limited mental health experience. Feedback interventions are typically developed from a professional perspective only, despite the acknowledged difference between professional and patient experiences or desires.^15^, ^28^, ^29^ The active involvement of patients in this research has therefore been imperative.

However, its limitations must also be acknowledged. Presented data represent a subsample of responses from one, although large, geographical area from one website. The need for further research in collaboration with patient‐research‐partners to explore potential cultural or demographic differences is therefore acknowledged. However, similar methodological restraints of single geographical areas are also reported in previous research,^18^ and should not undermine the practicality of the proposed framework. Other research limitations include the involvement of a small number of patient participants during the development stage, amalgamation of patient and carer perceptions, and an inability to assess original patient response satisfaction and motivation for providing patient feedback due to patient anonymity. Despite these limitations, as reported in previous research,^30^ it is anticipated that by developing and piloting the “PLLR” in a typically “hard‐to‐reach” population,^19^ the transferability of our research findings may be enhanced. While acknowledging the need for further research that addresses identified limitations, the conceptual framework proposed may also be applicable to other related fields outside of mental health due to their correlation with other literatures including business, hospitality and customer care.^9^, ^23^, ^31^

## 5 CONCLUSION

This research advances existing knowledge by collaboratively designing a patient feedback response framework from the patient perspective. It provides previously unavailable guidance on how to effectively respond to patient feedback online leading to clear practical and theoretical implications for those looking to listen to, learn from and respond to the patient voice in “real time.” By understanding patient perceptions of organizational responses, those responsible for developing and implementing response policies may be able to focus more precisely on factors considered important in effective organizational responses. This in turn could help health‐care services to develop more effective methods leading to enhanced response quality, patient safety and quality of care. To achieve this, organizations and providers must begin to align their response processes with patient aspirations and desires. By doing so, the invaluable learning opportunities attributed to patient experience can begin to be realized.

## CONFLICT OF INTEREST

The authors declare that they have no competing interests.
