# Supplementary material for: Responding effectively to adult mental health patient feedback in an online environment: A coproduced framework
Source: Health Expect. 2018 Apr 6;21(5):887–98. doi: 10.1111/hex.12682 (PMC6186539; doi:10.1111/hex.12682)
Supplement: Supplementary file 1 [file HEX-21-887-s001.docx]

| None provided | 62 |
| --- | --- |
| 1. PALS | 30 |
| 1. Patient experience manager | 12 |
| 1. Communications and engagement officer | 9 |
| 1. Communications manager | 5 |
| 1. PALS manager | 5 |
| 1. Director of nursing | 4 |
| 1. PALS and complaints lead | 4 |
| 1. Patient engagement administrator | 4 |
| 1. Patient engagement manager | 4 |
| 1. Development officer | 3 |
| 1. Head of clinical development | 3 |
| 1. Patient experience lead | 3 |
| 1. Volunteer, Healthwatch | 3 |
| 1. Advice and complaints team | 2 |
| 1. PALS and complaints manager | 2 |
| 1. Patient engagement lead | 2 |
| 1. Patient experience co-ordinator | 2 |
| 1. Advice and complaints team manager | 1 |
| 1. Business manager | 1 |
| 1. Clinical director | 1 |
| 1. Clinical governance | 1 |
| 1. Clinical team lead | 1 |
| 1. Co-founder and manager | 1 |
| 1. Communications and engagement manager | 1 |
| 1. Consultant nurse eating disorder | 1 |
| 1. Director of adult mental health | 1 |
| 1. Director of mental health and social care | 1 |
| 1. Head of communication | 1 |
| 1. Head of engagement and responsiveness | 1 |
| 1. Head of health, safety and security | 1 |
| 1. Head of inpatient services | 1 |
| 1. Head of patient experience | 1 |
| 1. Hospital director | 1 |
| 1. Lift | 1 |
| 1. Patient and Public experience lead | 1 |
| 1. Patient engagement and experience officer | 1 |
| 1. Patient experience and communications manager | 1 |
| 1. Patient experience team | 1 |
| 1. Perinatal service manager | 1 |
| 1. Senior quality facilitator | 1 |
| 1. Specialist nurse practitioner | 1 |

Additional file 1: Titles of responders identified
